# Production and Properties of Lignin Nanoparticles from Ethanol Organosolv Liquors—Influence of Origin and Pretreatment Conditions

**DOI:** 10.3390/polym13030384

**Published:** 2021-01-26

**Authors:** Johannes Adamcyk, Stefan Beisl, Samaneh Amini, Thomas Jung, Florian Zikeli, Jalel Labidi, Anton Friedl

**Affiliations:** 1 Institute of Chemical, Environmental and Bioscience Engineering, TU Wien, 1060 Vienna, Austria; stefan.beisl@tuwien.ac.at (S.B.); e1229284@student.tuwien.ac.at (S.A.); thomas.jung@tuwien.ac.at (T.J.); florian.zikeli@tuwien.ac.at (F.Z.); anton.friedl@tuwien.ac.at (A.F.); 2 Chemical and Environmental Engineering Department, University of the Basque Country UPV/EHU, 20018 Donostia-San Sebastián, Spain; jalel.labidi@ehu.eus

**Keywords:** lignin, nanoparticles, biorefinery, organosolv pretreatment

## Abstract

Despite major efforts in recent years, lignin as an abundant biopolymer is still underutilized in material applications. The production of lignin nanoparticles with improved properties through a high specific surface area enables easier applicability and higher value applications. Current precipitation processes often show poor yields, as a portion of the lignin stays in solution. In the present work, lignin was extracted from wheat straw, spruce, and beech using ethanol organosolv pretreatment at temperatures from 160–220 °C. The resulting extracts were standardized to the lowest lignin content and precipitated by solvent-shifting to produce lignin micro- and nanoparticles with mean hydrodynamic diameters from 67.8 to 1156.4 nm. Extracts, particles and supernatant were analyzed on molecular weight, revealing that large lignin molecules are precipitated while small lignin molecules stay in solution. The particles were purified by dialysis and characterized on their color and antioxidant activity, reaching ASC equivalents between 19.1 and 50.4 mg/mg. This work gives detailed insight into the precipitation process with respect to different raw materials and pretreatment severities, enabling better understanding and optimization of lignin nanoparticle precipitation.

## 1. Introduction

Lignin as the second most abundant biopolymer after cellulose has gained massive interest in recent years. It is estimated that approximately 2 × 10^11^ tons of lignocellulosic biomass residues are produced worldwide every year [1], making lignin a renewable phenolic compound available in large quantities. Since it is already produced in side-streams of pulp-productions and biorefineries, it has the potential to enable the transition from a fossil-based to a biobased economy by supplanting synthetic compounds currently produced from fossil resources. Since only around 40% of the lignin produced in pulping processes are needed to cover the internal energy demand of the processes [2,3], the possibility exists to vastly increase the amount of lignin used for material purposes providing efficient use of all biomass components and improving the sustainability of the process. Thus, the use of lignin as a material should be heavily expanded.

One challenge in the utilization of lignin is its diversity, as it is known to differ for different plant species, but also depending on the extraction process [4]. Lignin from the most common pulping processes, the Kraft and sulfite processes, contains a relatively-high amount of sulfur and may therefore not be suitable for many practical applications. In contrast, ethanol organosolv pretreatment yields lignin of high purity, with a structure closely resembling that of native lignin, and without sulfur contamination through the process [5,6], opening up possibilities for new applications [6]. A disadvantage of this process is its lower efficiency and high energy demand for solvent recovery [7]. Improvement of the process economy while still maintaining the high quality of the lignin produced will be necessary to compete with other pretreatment technologies on the one hand and fossil-based products that are aimed to be replaced by lignin on the other.

Although increased process severity tends to increase delignification and lignin concentration in the liquor [8], which would be desirable for increased process efficiency, simultaneously more sugar degradation products are formed [9], and the lignin changes structurally, which might be problematic for the final product. Thus, process efficiency needs to be balanced out with the final product quality.

Another possibility to improve the economy of a biorefinery based on organosolv pretreatment is finding high value applications for the produced lignin. Recent works have shown that colloidal lignin particles have many desirable and improved properties which lead to a wide range of possible applications [10], due to the high ratio of surface to volume. Especially the application of lignin for UV-protection in cosmetics [11,12,13] but also wood conservation [14] has been reported and commercial use in these areas seems within reach. Qian et al. [15] reported that the ability to block UV-radiation is improved for smaller particle sizes, which underlines the connection between improved properties and particle size. However, previous works have shown that a major portion of the lignin remained in solution after the precipitation of colloidal particles, resulting in a lower overall yield [16,17]. This raises the suspicion that lignin gets fractionated by molecular weight in the precipitation step, which was confirmed for commercial soda lignin by Sipponen et al. [18]. Further understanding of the precipitation of lignin should make it possible to improve the process towards higher yields while still maintaining small particle sizes.

Based on this, the present work investigates the extraction with ethanol organosolv pretreatment and the subsequent precipitation of lignin into colloidal particles. Three different raw materials (wheat straw, spruce wood, and beech wood), representing three combinations of guaiacyl (G), *p*-hydroxy phenol (H) and syringyl (S) lignins common in nature (GSH, G and GS lignins, respectively), and four temperatures (160–220 °C) were applied in the pretreatment process. Through this experimental plan, the influence of raw material and process severity on the lignin precipitation was studied. The extracts’ compositions were characterized, and the prepared lignin particles were analyzed regarding their size and physico-chemical properties. The different process fractions were analyzed regarding their molecular weight to further investigate the precipitation process.

## 2. Materials and Methods

**Figure 1:**
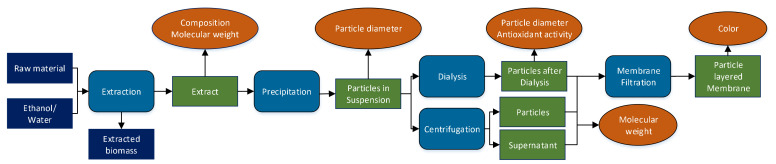
Schematic of the experimental plan conducted for each pretreatment condition and raw material. The analyzed process fractions are depicted in green.

An overview of the experimental procedure is shown in Figure 1, indicating process steps, fractions, and analytics. Generally, lignin was extracted from the different raw materials and precipitated into colloidal particle suspensions by addition of an antisolvent. A part of these suspensions was centrifuged for analytics, the remainder was dialyzed to remove dissolved impurities. The last step was membrane filtration to produce a thin particle layer for color measurements.

The particle fraction separated via dialysis is termed “particles after dialysis” and the particle fraction separated via centrifugation is termed “particles” throughout the whole manuscript. The particles after the centrifugation are considered the same as the ones in the suspension directly after precipitation.

### 2.1. Materials

The wheat straw used was harvested in 2015 in Lower Austria, the spruce wood was supplied by Laakirchen Papier (Laakirchen, Austria), the beech wood by Lenzing AG (Lenzing, Austria). The particle size of all raw materials was reduced in a cutting mill with a 2 mm mesh, the resulting material was stored under dry conditions until the pretreatment. Ultra-pure water (18 MΩ/cm) and Ethanol (Merck, 96 vol %, undenaturated, Darmstadt, Germany) was used in the organosolv treatment.

### 2.2. Pretreatment/Extraction

The organosolv pretreatment was conducted in a 1 L stirred autoclave (Zirbus, HAD 9/16, Bad Grund, Germany) using a 60 wt% aqueous ethanol mixture as solvent under consideration of the water content in the raw material. The biomass content in the reactor based on dry matter was 8.3 wt%. The reactor was heated to the pretreatment temperature and then held at this temperature. After 60 min of total pretreatment time, the reactor was cooled to room temperature. The solid and liquid fractions were separated using a hydraulic press (Hapa, HPH 2.5, Achern, Germany) at 200 bar, the extract was then centrifuged (Thermo Scientific, Sorvall, RC 6+, Waltham, MA, USA) at 24,104× *g* for 20 min to remove residual solids. The particle-free extracts of the single batches were unified, the composition was analyzed, and it was stored at 5 °C until the precipitation experiments were performed.

### 2.3. Precipitation

To eliminate the influence of the lignin concentration on the precipitation, the concentration of all extracts was set to the lowest total lignin concentration (3.52 g/L) by dilution with 60 wt% aqueous ethanol. The precipitation experiments were conducted at 25 °C, the volume ratio of extract to antisolvent was kept constant at 1:5, where the antisolvent consisted of Ultra-pure water. Setup (b) as described in [16] was used for the precipitations. The resulting suspensions were stored at 5 °C.

### 2.4. Downstream Processing

Parts of the suspensions were centrifuged in a Thermo WX Ultra 80 ultracentrifuge (Thermo Scientific, Waltham, MA, USA) at 288,000× *g* for 60 min. The supernatants were decanted and stored at 5 °C, the liquid free particles were freeze dried and stored in a desiccator.

The rest of the suspensions were dialyzed for 7 days in excess of water (daily replaced) using Nadir^®^ dialysis hoses with 10,000–20,000 Dalton cut-off. The particles after dialysis were stored at 5 °C.

### 2.5. Analytics

#### 2.5.1. Extract Characterization

The organosolv extract was analyzed for carbohydrates, lignin, and degradation products. The carbohydrate content was determined by the sample preparation following the National Renewable Energy Laboratory (NREL) laboratory analytical procedure (LAP) “Determination of sugars, byproducts, and degradation products in liquid fraction process samples” [19] but with no neutralization of the samples after hydrolysis. A Thermo Scientific ICS-5000 HPAEC system equipped with a photo array detector (PAD) with deionized water as the eluent was used for the determination of arabinose, glucose, mannose, xylose, and galactose. The concentrations of the degradation products acetic acid, hydroxymethylfurfural (HMF) and furfural were determined with a Shimadzu LC-20A “prominence” HPLC system and a Shodex SH1011 (Showa Denko, Tokyo, Japan) column at 40 °C, with 0.005 M H_2_SO_4_ as eluent. The acid insoluble and acid soluble lignin contents were determined following the NREL LAP “Determination of Structural Carbohydrates and Lignin in Biomass” [20] using the dry matter of the extract obtained at 105 °C.

#### 2.5.2. HP-SEC Analysis

Molar mass distributions of the standardized organosolv extracts, suspensions, centrifuged lignin particles, supernatants, and lignin particles after dialysis were determined by alkaline High Performance Size Exclusion Chromatography (HP-SEC) analysis (eluent: 10 mM NaOH) using three TSK-Gel columns in series at 40 °C (PW5000, PW4000, PW3000; TOSOH Bioscience, Darmstadt, Germany) with an Agilent 1200 HPLC system (flow rate: 1 mL/min, DAD detection at 280 nm, Santa Clara, CA, USA). Solid lignin fractions were dissolved in the eluent, and liquid lignin fractions were diluted with the eluent to adjust the pH for analysis. Calibration of the columns set was done using polystyrene sulfonate reference standards (PSS GmbH, Mainz, Germany). The molar masses at peak Maximum (M_p_) were 78,400 Da, 33,500 Da, 15,800 Da, 6430 Da, 1670 Da, 891 Da, and 208 Da.

#### 2.5.3. Hydrodynamic Diameter

Particle size measurements were conducted in a ZetaPALS (Brookhaven Instruments, Holtsville, NY, USA) using dynamic light scattering (DLS). The refractive index of the particles was set to 1.53 and the imaginary refractive index to 0.1. All reported mean values for hydrodynamic diameters are based on intensity based distributions.

#### 2.5.4. Color

The colorimeter PCE-CMS 7, PCE Instruments (Albacete, Spain) was used to measure the Hunter color values (L, a, and b) of the lignin. The lignin particles after dialysis were filtered against a HFK™-131 ultrafiltration membrane (Koch Membrane Systems, Rimsting, Germany) resulting in layer densities of lignin ranging from around 1 g/m^2^ to 14 g/m^2^. All membranes were purged with 10 wt% aqueous ethanol and the color background (L = 99.08, a = 0.001, b = −4.911) was measured after purging. The color of each lignin sample was measured at three different lignin layer densities and was standardized to 3 g/m^2^ via linear regression of the L, a, and b values.

#### 2.5.5. Antioxidant Activity

All particle suspensions were filtrated using a PES-Filter (Merck, Darmstadt, Germany) with a pore size of 0.22 µm to remove agglomerates and yield comparable particle size distributions.

*ABTS:* The determination of 2,2′-azinobis-(3-ethylbenzothiazoline-6-sulfonic acid) (ABTS) radical inhibition was based on the method of Re et al. [21]. Twenty-five milliliters of ABTS stock solution was prepared in dark bottle by dissolving 69.7 mg of ABTS salt and 11.8 mg K_2_S_2_O_8_ in water. The stock solution was left in a refrigerator and diluted with deionized water to obtain an initial absorbance of 0.7 at 734 nm in a Jasco V-630 spectrophotometer (JASCO, Tokyo, Japan) before use. To determine the radical scavenging ability, the sample was added to the diluted ABTS solution. The absorbance was measured after 6 min incubation at 734 nm. The percentage of inhibition was calculated from below equation:

AA[%] = (Ac − At)/Ac × 100%(1)

where: AA-antioxidant activity, Ac—absorbance of control sample, At—absorbance of tested sample.

*FRAP:* The ferric reducing antioxidant power (FRAP) test is based on the reduction of Fe(III) to Fe(II) by the antioxidant compound which forms a colored complex (593 nm) with 2,4,6-tripyridyl-s-triazine (Fe(II)-TPTZ) in acetate buffer at pH 3.6 [22]. Briefly, the reactive solution was freshly prepared with 25 mL of 300 mM acetate buffer (pH 3.6), 2.5 mL of 10 mM 2,4,6-tripyridyl-s-triazine in 40 mM HCl and 2.5 mL 20 mM FeCl_3_·6H_2_O in distilled water. Samples (0.1 mL) were mixed with 3 mL of the FRAP reactive solution. The reference standard used was ascorbic acid (ASC) and results are given as ASC equivalents.

## 3. Results

### 3.1. Extract Composition

**Figure 2:**
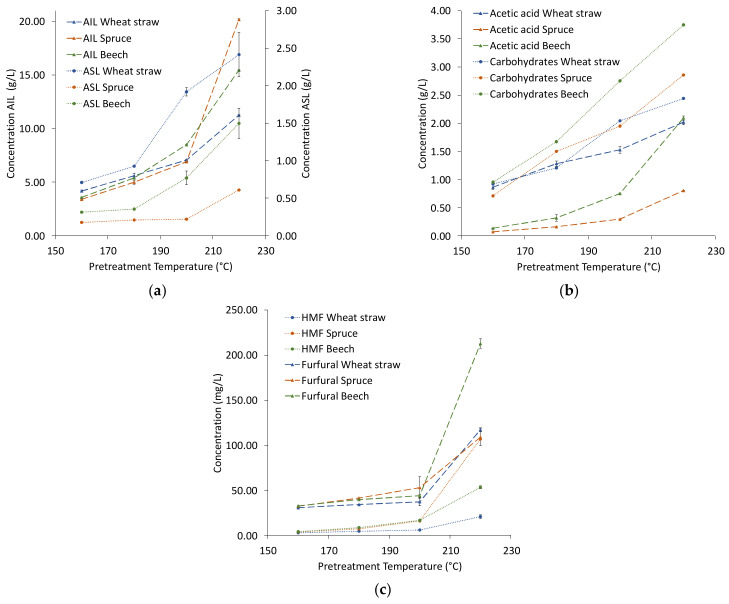
Concentration of lignin (**a**), acetic acid and carbohydrates (**b**), and degradation products (**c**) in organosolv extracts. Abbreviations: AIL—acid insoluble lignin, ASL—acid soluble lignin, HMF—hydroxymethylfurural.

The composition of the extracts is shown in Figure 2. The amount of each compound in the extract increases with temperature, the largest increase of acid insoluble lignin (AIL) and the degradation products happens from 200 to 220 °C for all raw materials, indicating a considerable increase in process severity. Lignin is the dominant compound in the dry matter of the extracts, making up between 50 wt% for extracts from straw and 70 wt% for extracts from the woods. From 160 to 200 °C, the total lignin content of the wheat straw extracts is higher than that of the woods due to the consistently higher amount of acid soluble lignin (ASL) in wheat straw extracts, while at 220 °C the lignin content in the extract from spruce is highest, followed by beech (Figure 2a). This could suggest that although the initial lignin content of wheat straw is lower than that of the woods, the extraction is more efficient already at lower temperatures, and a higher severity is required for efficient delignification of the woods when using ethanol organosolv pretreatment. Siika-aho et al. [23] applied an oxygen/carbonate process to three different raw materials at varying process severities and also found delignification of straw to be more efficient at low process severities compared to spruce and beech.

The trends of the carbohydrates in the extracts (Figure 2b) are similar for all raw materials, though beech wood has consistently the highest concentrations. For acetic acid, extraction from wheat straw results in a higher concentration than from the woods in most cases, though there is a strong increase for beech from 200 °C to 220 °C. For the degradation products (Figure 2c), the amounts are of the same order of magnitude from 160 °C to 200 °C, at 220 °C hydroxymethylfurfural (HMF) of spruce and furfural of beech stand out.

**Figure 3:**
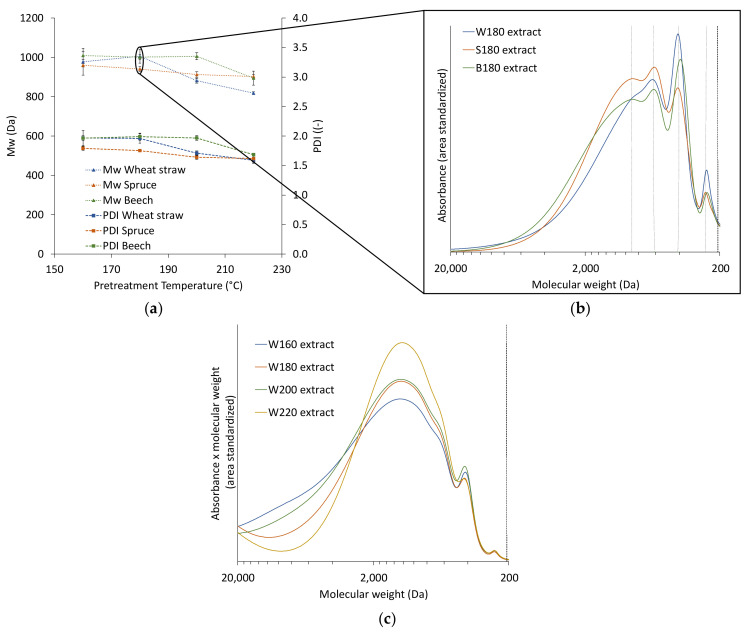
M_w_ and PDI of the extracts from the different raw materials (**a**), molecular weight distribution of extracts from different raw materials at 180 °C (**b**), and molecular weight distribution of wheat straw at different pretreatment temperatures (**c**). Abbreviations: W—wheat straw, S—spruce, B—beech; the number next to the letter is the temperature of the corresponding pretreatment temperature.

To remove the influence of the lignin concentration in the precipitation step, the extracts were diluted to the lowest lignin concentration (3.52 g/L) with aqueous ethanol (60 wt %). As demonstrated in Figure 3a, the molecular weight distribution of the lignin in the extracts is influenced by the pretreatment temperature and the raw material. The changes between the raw materials become more visible in the molecular weight distributions (Figure 3b). There are four distinct peaks of different molecular weight, with maxima at approximately 900, 610, 410, and 255 Da. The two lower molecular weight fractions are more dominant in the wheat straw extracts than in those of the woods, which could be linked to the higher concentration of ASL found in wheat straw extracts. In earlier works the third peak was found to be strongly influenced by p-hydroxycinnamic acids like ferulic acid and p-coumaric acid, in monomeric form or connected to lignin fragments present in wheat straw [24]. Apart from the differences in the raw materials, the pretreatment temperature influences the molecular weight distribution of the dissolved lignin; large, dissolved lignin molecules are depolymerized at higher temperatures, and lignin fragments can again re-condensate at severe conditions [25]. The combination of raw material and temperature decides which effect is dominant. The M_w_ of all three raw materials show a trend to lower values with increasing temperature (Figure 3b), which is more pronounced for wheat straw than for the woods. In Figure 3c, the absorbance is multiplied with the molecular weight to highlight the strong influence of high molecular weight fractions on the M_w_. These molecular weight distributions also indicate (stronger than the weight averaged values) that the temperature increase from 200 to 220 °C significantly influences the molecular weight.

### 3.2. Precipitation

The lignin dissolved in the extracts was precipitated into micro- and nanoparticles by addition of water as an antisolvent. The resulting suspensions were characterized on the particles’ hydrodynamic diameter by dynamic light scattering. The particles and supernatant were separated by centrifugation for analysis; parts of the suspensions were dialyzed to remove impurities before further analytics. Visible agglomerates formed during dialysis were removed by filtration before determination of the antioxidant activity in order to analyze exclusively colloidal particles. Molecular weight of lignin particles after precipitation, dissolved lignin in the supernatants, and lignin particles after dialysis was measured. The yields of the precipitations could not be determined due to too low sample amounts.

**Figure 4:**
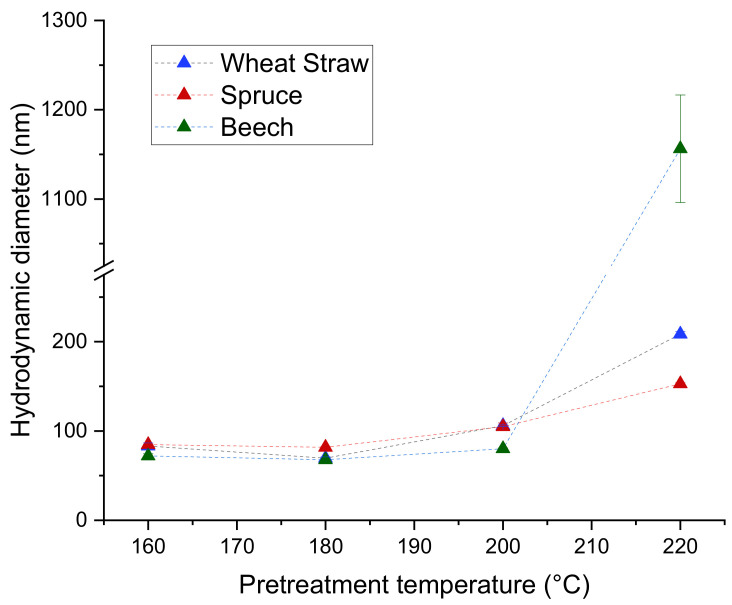
Hydrodynamic diameters of lignin particles.

The hydrodynamic diameter of the particles stays relatively similar for each raw material between 160 °C and 200 °C, with only a slight trend upwards and diameters between 67.8 nm and 105.9 nm. However, the extracts from 220 °C yielded significantly bigger particles with diameters ranging from 152.8 nm for spruce to 1156 nm for beech (Figure 4), suggesting that the precipitation is strongly influenced by changes in the lignin occurring above 200 °C of pretreatment temperature.

**Figure 5:**
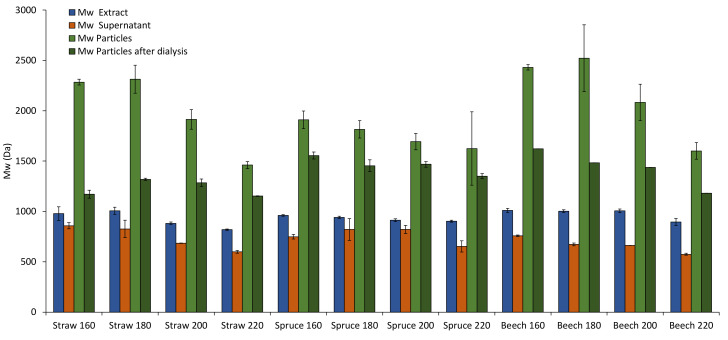
M_w_ in the different process fractions for wheat straw, spruce, and beech.

**Figure 6:**
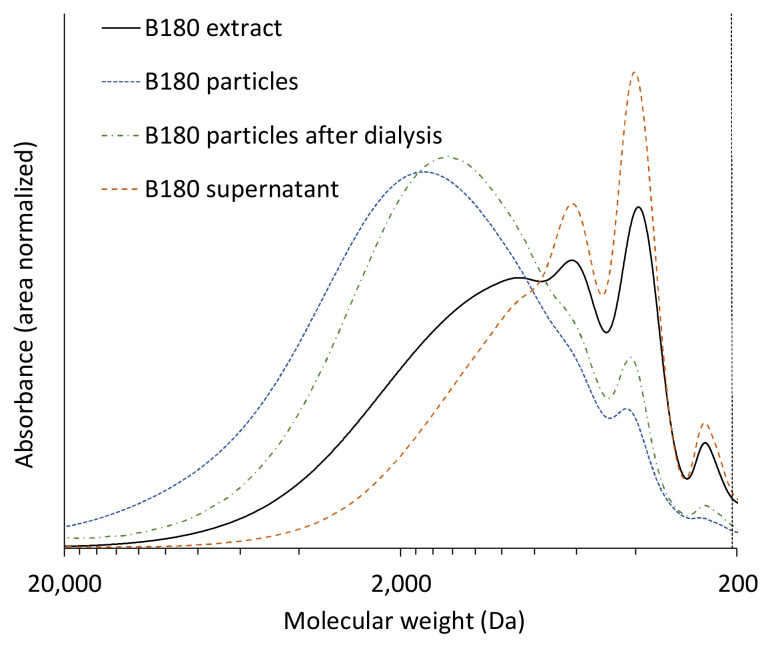
Molecular weight distributions of extract, particles, particles after dialysis, and supernatant from beech wood experiments at 180 °C pretreatment temperature.

This leads back to the differences in molecular weight distribution found in the extracts for different pretreatment temperatures and raw materials. In Figure 5, the M_w_s of all raw materials, pretreatment temperatures and fractions are presented. The results show that M_w_s of the lignin in the extracts are lower than those of the particles as well as of the particles after dialysis, but higher than those of the supernatants for all experiments. This supports the assumption that during the solvent shifting precipitation mainly lignin of high molecular weight is precipitated into particles while lignin of lower molecular weight remains in solution. Figure 6 showcases this fractionated precipitation for beech wood pretreated at 180 °C. Sipponen et al. [18] also found this molar-mass-fractionation when precipitating wheat straw soda lignin from aqueous ethanol by water addition and attributed it to electronic interactions of the aromatic lignin structures, which lead to lower solubility of larger lignin molecules.

Interestingly, the M_w_s of the particles separated from the suspension directly after precipitation are consistently higher than those of the particles separated after dialysis (light and dark green columns in Figure 5). This indicates that some lignin with lower molecular weight is still in solution after precipitation but not removed by dialysis, despite a much higher membrane molecular weight cut-off. A possible explanation for this is that the gradual ethanol removal during dialysis lowers the lignin solubility. Smaller lignin molecules that are dissolved in the supernatant would precipitate onto the existing particles due to this, increasing their diameter and facilitating agglomeration. The results show that a portion of the lignin dissolved in the supernatant is removed by dialysis, since the M_w_ of the particles after dialysis is always higher than that of the extract.

**Figure 7:**
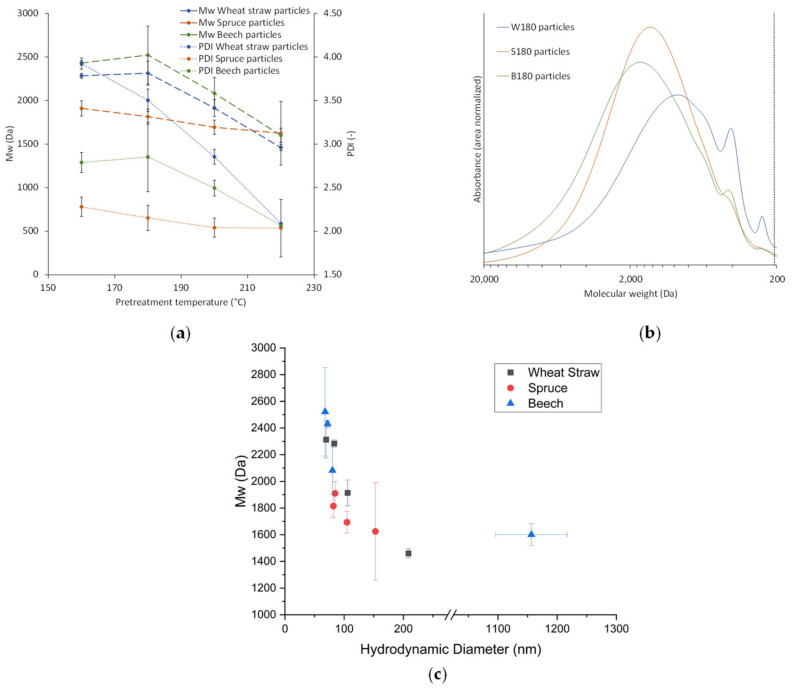
M_w_ and PDI of lignin particles (**a**), molecular weight distribution of particles from different raw materials at 180 °C pretreatment temperature (**b**), M_w_ of particles over diameter of particles (**c**).

The trend to lower M_w_s at higher pretreatment temperatures found in the extracts (Figure 3a) is also present and more pronounced for the particles (Figure 5), which coincides with the increase in particle size at higher temperatures (Figure 4). A possible explanation for this is that at higher pretreatment temperatures more lignin is depolymerized into smaller molecules which have a higher solubility than the larger molecules in the same extract. Because of the higher solubility of smaller lignin molecules, the degree of local supersaturation after the mixing would be lower for extracts produced at 220 °C, causing slower precipitation and larger particles [26]. Figure 7c shows this decrease of particle size with increasing M_w_. Zwilling et al. [27] also found this increase of particle size with decreasing molecular weight, and connected it with more hydrophilic character of low molecular weight lignin. However, in this work lignin structure was not investigated.

Comparison of the particles’ molecular weight distributions for the different raw materials (Figure 7) shows that spruce wood extracts yield particles with lower M_w_s and PDIs than wheat straw or beech wood extracts. Figure 7b exemplifies this and additionally shows that the lignin particles from the woods have a similar distribution, while wheat straw results in a much broader distribution with more dominant peaks at 410 Da and 210 Da. At 220 °C, M_w_s and PDIs of all raw materials are within the same range and generally lower, suggesting that the lignin of all raw materials is depolymerized to a similar degree (Figure 7a). Comparing Figure 3a and Figure 7a shows that the differences between the M_w_s or PDIs found for the particles are not present to the same extent in the respective extracts, which means that the precipitation amplifies differences between lignin from different raw materials. This might in turn have an influence on the particles’ properties.

### 3.3. Lignin Properties

Since the particle size generally increased strongly for the precipitations from 220 °C extracts, and the precipitation did not give reasonable results for beech at that temperature, the color and antioxidant potential were only determined for lignin particles after dialysis from pretreatment temperatures 160 °C to 200 °C.

#### 3.3.1. Color

**Figure 8:**
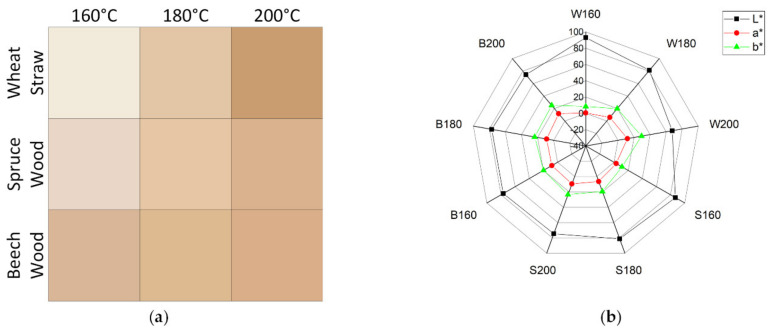
Color of a 3 g/m^2^ layer of lignin particles (**a**) and affiliated components in CIELAB-color space (**b**).

For many of the possible commercial material applications of colloidal lignin particles, like in sunscreens or food packaging [10], it is important to consider the color of the produced lignin particles. For example, if lignin particles are being used in a product for their antioxidant activities to avoid color change in a product, a dark, brown particle color is obstructive to the application and a limiting factor to the maximum particle concentration applicable. Figure 8 shows the colors of equally thin layers of lignin particles on a membrane and the respective values in the CIELAB-color space. Higher pretreatment temperatures lead to darker lignin and stronger colors, demonstrated by the lowered brightness (L *) and increased reddening (+a *) and yellowing (+b *) values in the spider graph, which can be considered undesirable. The darkening of the lignin particles can be attributed to increase of condensation reactions at higher process severities. The color changes most for wheat straw and least for beech wood with increasing pretreatment temperature. The ratio of red to yellow color component is similar for all lignins, except for wheat straw at 160 °C, where the yellow component is more dominant. Generally, through variation of raw material and pretreatment temperature a wide array of colors in the brown spectrum can be achieved for lignin particles. The influence of the particle size on the color found by other groups [28] could not be investigated with the given experimental plan, since too many other contributing factors were varied, and the investigated particles where of similar diameters.

#### 3.3.2. Antioxidant Activities

**Figure 9:**
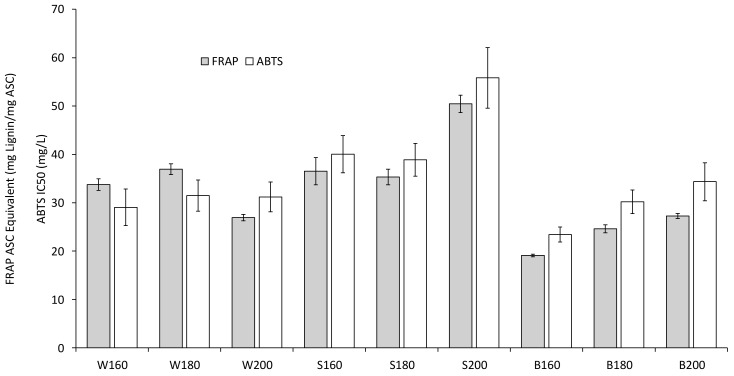
Antioxidant activities of particles after dialysis.

Before determining the antioxidant activities of the particles after dialysis, agglomerates and large particles were removed from the particle suspensions after dialysis by filtration, which resulted in suspensions with hydrodynamic diameters between 65.7 and 90.7 nm. Both methods used to determine the antioxidant activity (FRAP and ABTS) show similar trends (Figure 9): Higher pretreatment temperatures lead to lower antioxidant activity for beech, for spruce this effect is only witnessed when the temperature is increased from 180 to 200 °C. For straw, the antioxidant activity does not significantly change with the temperature for ABTS, for FRAP it reaches a maximum at 200 °C. The antioxidant potential of the beech lignin particles is highest at 160 and 180 °C according to both methods, at 200 °C wheat straw reaches similar values. The divergence in the results between ABTS and FRAPS makes it difficult to compare the antioxidant potential of particles from spruce and wheat straw at 160 and 180 °C.

It is important to note that the antioxidant potential was measured for the particles (not solubilized lignin), and therefore is also affected by the surface area. Zhang et al. [29] investigated the antioxidant activities of lignin nanoparticles from corncob residue and found IC_50_ values between 101.2 and 296.6 mg/L. Due to the variation in raw materials and pretreatment temperatures combined with comparatively small differences in the particle diameters, the influence of the particle size on the antioxidant activity found in other works could not be investigated in this work.

## 4. Conclusions

The production and properties of colloidal lignin particles from organosolv extracts from different raw materials and the influence of the pretreatment temperature was investigated in the present work. All investigated raw materials (wheat straw, spruce wood, beech wood) were successfully applied for the production of colloidal particles with diameters below 110 nm whereas the origin of the lignin showed only minor influence in the size of the resulting particles.

Higher pretreatment temperatures increase the delignification of the raw materials but also favor depolymerization and structural alteration of the extracted lignin. This leads to increased particle sizes of over 110 nm and agglomeration at pretreatment temperatures of over 200 °C.

The investigation of the precipitation via solvent-shifting showed lignin fractionation by molecular weight, meaning that large lignin molecules precipitate while smaller lignin molecules stay in solution.

The resulting lignin nanoparticles were characterized on their application relevant properties of color and antioxidant potential. Higher pretreatment temperatures generally results in particles with darker colors and lower antioxidant activity especially for particles produced from beech and spruce. The antioxidant activity of the particles ranged from 19.1 and 50.4 mg Lignin/mg ASC equivalents.

From a process perspective, it is desirable to reach a high concentration of lignin in the extract, which can be achieved by higher pretreatment temperatures. However, this increase in process efficiency comes at the cost of lignin quality, which is demonstrated by the sharp increase in particle diameter, darker color of the resulting particles, and the decrease in antioxidant activity of the lignin particles. Therefore, optimal conditions and choice of raw material in a commercial lignin nanoparticle production process heavily depend on the final application.

## Data Availability

The data presented in this study are available on request from the corresponding author.
